# Supplementary figures and images for: The Envelope Proteins from SARS-CoV-2 and SARS-CoV Potently Reduce the Infectivity of Human Immunodeficiency Virus type 1 (HIV-1)
Source: Res Sq. 2022 Oct 24:rs.3.rs-2175808. Preprint. [Version 1] doi: 10.21203/rs.3.rs-2175808/v1 (PMC9628187; doi:10.21203/rs.3.rs-2175808/v1)

## Slide 1
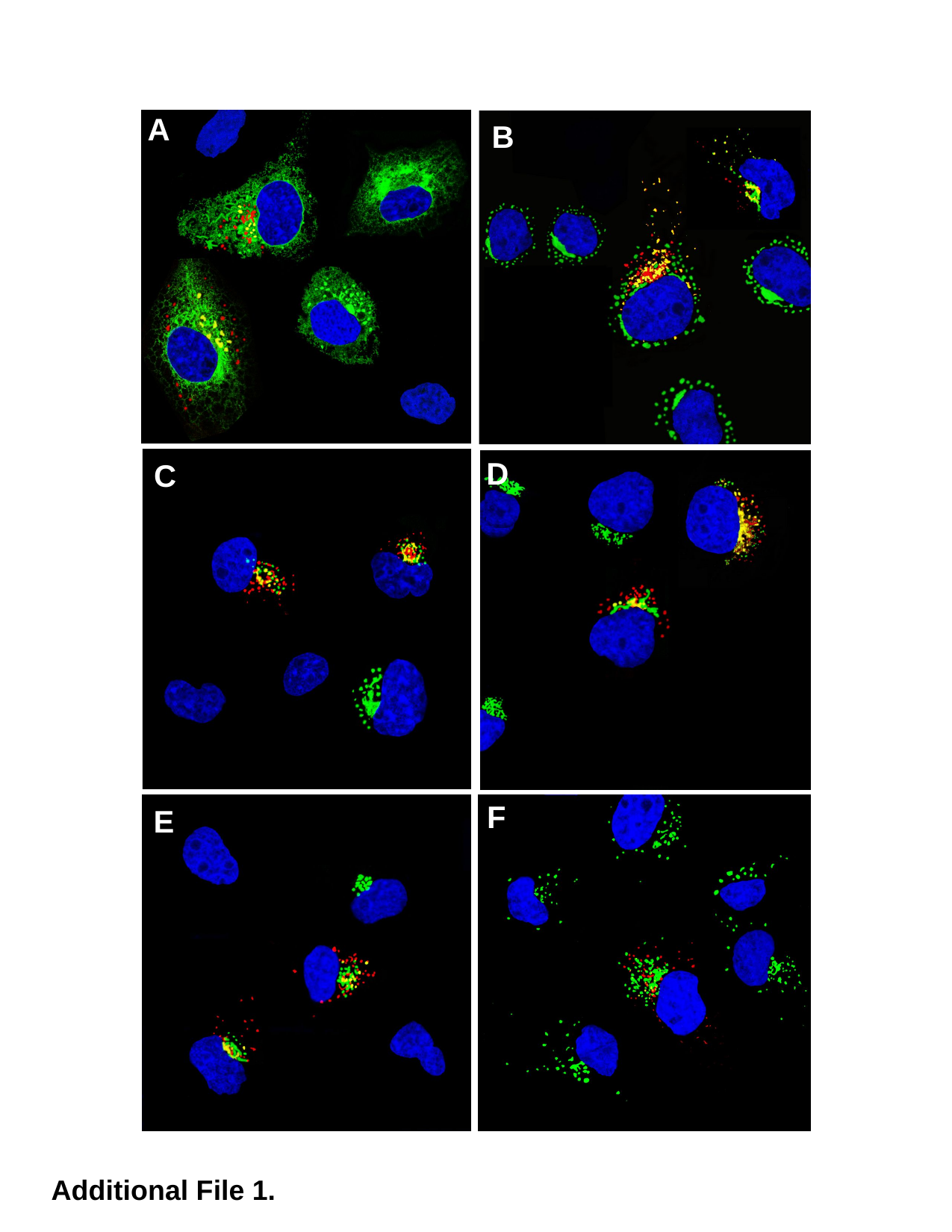

A
B
D
C
F
E
Additional File 1.

Supplement: Supplement 1 [file Additional_file_1.pptx]

## Slide 1
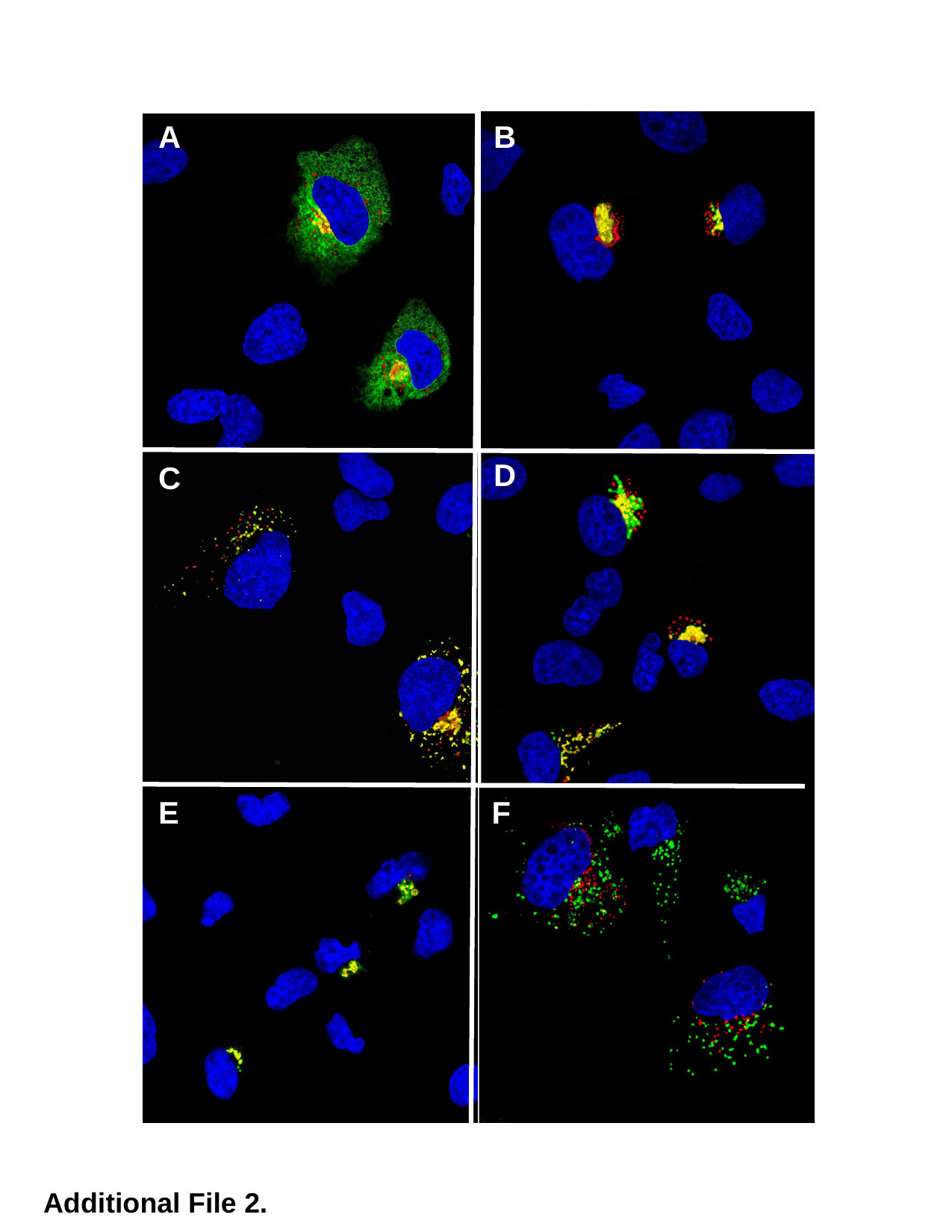

A
B
D
C
E
F
Additional File 2.

Supplement: Supplement 2 [file Additional_file_2.pptx]
